# Overexpression of *AtOxR* gene improves abiotic stresses tolerance and vitamin C content in *Arabidopsis thaliana*

**DOI:** 10.1186/s12896-016-0299-0

**Published:** 2016-10-07

**Authors:** Yuanyuan Bu, Bo Sun, Aimin Zhou, Xinxin Zhang, Testuo Takano, Shenkui Liu

**Affiliations:** 1 Key Laboratory of Saline-Alkali Vegetation Ecology Restoration in Oil Field (SAVER), Ministry of Education, Alkali Soil Natural Environmental Science Center (ASNESC), Northeast Forestry University, Harbin, 150040 People’s Republic of China; 2 Heilongjiang River Fisheries Research Institute, Chinese Academy of Fishery Sciences, Rd 232 Hesong, Daoli District, Harbin, 150070 China; 3 Asian Natural Environmental Science Center(ASNESC), The University of Tokyo, Nishitokyo, Tokyo 188-0002 Japan

**Keywords:** Abiotic stresses, L-ascorbic acid (AsA, Vitamin C), Hydrogen peroxide (H_2_O_2_), Transgenic plants

## Abstract

**Background:**

Abiotic stresses are serious threats to plant growth, productivity and result in crop loss worldwide, reducing average yields of most major crops. Although abiotic stresses might elicit different plant responses, most induce the accumulation of reactive oxygen species (ROS) in plant cells leads to oxidative damage. L-ascorbic acid (AsA, vitamin C) is known as an antioxidant and H_2_O_2_-scavenger that defends plants against abiotic stresses. In addition, vitamin C is also an important component of human nutrition that has to be obtained from different foods. Therefore, increasing the vitamin C content is important for improving abiotic stresses tolerance and nutrition quality in crops production.

**Results:**

Here, we show that the expression of *AtOxR* gene is response to multiple abiotic stresses (salt, osmotic, metal ion, and H_2_O_2_ treatment) in both the leaves and roots of *Arabidopsis*. AtOxR protein was localized to the Endoplasmic Reticulum (ER) in yeast and *Arabidopsis* cells by co-localization analysis with ER specific dye. AtOxR-overexpressing transgenic *Arabidopsis* plants enhance the tolerance to abiotic stresses. Overexpression of *AtOxR* gene resulted in AsA accumulation and decreased H_2_O_2_ content in transgenic plants.

**Conclusions:**

In this study, our results show that AtOxR responds to multiple abiotic stresses. Overexpressing AtOxR improves tolerance to abiotic stresses and increase vitamin C content in *Arabidopsis thaliana*. AtOxR will be useful for the improvement of important crop plants through moleculer breeding.

**Electronic supplementary material:**

The online version of this article (doi:10.1186/s12896-016-0299-0) contains supplementary material, which is available to authorized users.

## Background

In natural environments, plant growth and crop productivity are reduced by abiotic stresses such as high salinity, drought, heavy metals, and oxidative stress. Although these stresses might elicit different plant responses, most induce the accumulation of reactive oxygen species (ROS) [1, 2], including hydroxyl radicals (OH^−^), superoxide anions (O_2_^−^), and hydrogen peroxide (H_2_O_2_). The production of large amounts of ROS in plant cells leads to oxidative damage [3–5]. In our previous study, we prepared a cDNA library from seedlings of a salt-tolerant plant, *Puccinellia tenuiflora* that had been treated with 150 mM NaHCO_3_ [6]. One of the sequenced genes, *PutOxR*, was found to confer enhanced tolerance to multiple abiotic stresses in yeast. *Arabidopsis thaliana* has a homologous gene, which in our preliminary studies was found to be associated with multiple abiotic and oxidative stress. We thus named it AtOxR (GenBank accession No: NP_568854), furthermore, the study of *AtOxR* gene have not been reported.

H_2_O_2_-scavenging in plants is achieved through several mechanisms, for example, the water–water cycle, catalase (CAT) enzymatic reactions, the glutathione peroxidase (GPX) cycle, and the ascorbate-glutathione (GSH) cycle [3]. These pathways involve a number of enzymes, such as superoxide dismutase (SOD), ascorbate peroxidase (APX), Catalase (CAT), and glutathione peroxidase (GPX) [7–9], and anti-oxidants such as glutathione [10, 11] and ascorbic acid (AsA) [10, 12]. In plants, H_2_O_2_ levels can be changed via the AsA/glutathione (GSH) cycle, APX plays a central role in the cycle and is emerging as a key enzyme in cellular H_2_O_2_ metabolism.

AsA is not only an important component of human nutrition but also an antioxidant and H_2_O_2_-scavenger that defends plants against abiotic stresses [13–16]. For example, enhanced AsA accumulation confers tolerance to oxidative, salt and drought stresses in potato and maintenance of a high AsA level is required for oxidative stress tolerance in *Arabidopsis* [17, 18]. AsA is synthesized through multiple biosynthetic pathways in plants [19–22], while the major pathway is Smirnoff–Wheeler pathway [22, 23]. Previous studies have shown that overexpression of AsA biosynthetic pathway related genes resulted in increased vitamin C content [24–30]. In addition, co-expression of *NCED* and *ALO* improves vitamin C content and tolerance to abiotic stresses in transgenic tobacco and stylo plants [31]. Although vitamin C is essential, humans are one of the few mammalian species unable to synthesis the vitamin and must obtain it through dietary sources. In recent years, the physiological function that AsA plays in abiotic stress tolerance and nutritional quality has garnered increasing attention.

In the present study, the expression pattern of *AtOxR* in seedlings treated by abiotic stresses was studied using quantitative real-time PCR (qRT-PCR). The subcellular localization of the AtOxR protein and the phenotypes of *AtOxR* gene transformants in yeast and *Arabidopsis* were also analyzed. Additionally, the H_2_O_2_ and vitamin C content of *AtOxR* transgenic *Arabidopsis* and WT plants were determined in plants grown under abiotic stresses.

## Methods

### Plasmid constructs and plant materials

The open reading frame (ORF) of AtOxR was amplified from *Arabidopsis* cDNA using the primers AtOxR-FW and AtOxR-RV (Table 1). The amplified product AtOxR was digested with *Kpn*I and *Bam*HI and cloned into the yeast expression vector pYES2 (Invitrogen) to form pYES2-AtOxR plasmid, which was transferred into *Saccharomyces cerevisiae* strain InVsc1. For the construction of GFP fusion proteins, AtOxR without its stop codon was amplified with the primers AtOxR-FW-*Bam*HI and AtOxR-RV-*Kpn*I (Table 1) by using AtOxR cDNA as a template, the amplified product was digested with *Bam*HI and *Kpn*I, and cloned into the pEGFP vector (Invitrogen). The construct plasmid pEGFP-AtOxR-GFP was digested with *Bam*HI and *Eco*RI, and cloned into the pYES2 vector to obtain the plasmid pYES2-AtOxR-GFP, which was transferred into InVsc1 using a lithium acetate-based method [32]. AtOxR-GFP was amplified from pEGFP-AtOxR-GFP with primers AtOxR-FW-*Bam*HI and GFP-RV-*Sac*I (Table 1). The product was then digested with *Bam*HI and *Sac*I, and cloned into the pBI121 vector to obtain the plasmid pBI121-AtOxR-GFP. The constructs pBI121-AtOxR, pBI121-AtOxR-GFP and pBI121-GFP as control were transformed into *Agrobacterium tumefaciens* strain EHA105 to obtain the transgenic *Arabidopsis* by the floral dip method [33].

**Table 1:** Sequence of the primers used for PCR

Primer	Sequence (5’- 3’)
*AtOxR-FW*	ATGTATTTCGCCGCCATAGC
*AtOxR-RV*	TAAGCCTCTCTCCGTTTTCTCC
*pBI121-AtOxR-FW*	GGATCCTATGTATTTCGCCG *(BamHI)*
*pBI121-AtOxR-RV*	GAGCTCCTCCTCTAATAGTC *(SacI)*
*AtOxR-FW-BamHI*	GGTACCATGTATTTCGCCGCCATAGC *(BamHI)*
*AtOxR-RV-KpnI*	GGATCCGCCTCTCTCCGTTTTCTCC *(KpnI)*
*GFP-RV-SacI*	GAGCTCCTAGAGTCGCGGCCGC *(SacI)*
*pYES2-AtOxR-FW*	GGTACCTATGTATTTCGCCG *(KpnI)*
*pYES2-AtOxR-RV*	GGATCCCTCCTCTAATAGTC *(BamHI)*
*AtOxR-RT-FW*	TACGGAGGTTACGGATGGTC
*AtOxR-RT-RV*	GCCTCTCTCCGTTTTCTCCT
*Actin-FW*	GGTAACATTGTGCTCAGTGGTGG
*Actin-RV*	AACGACCTTAATCTTCATGCTGC

### Phylogenetic analyses, yeast transformations, and growth conditions

Full-length amino acids sequences were aligned using ClustalX, and then imported into the Molecular Evolutionary Genetics Analysis (MEGA) package version 3.1 [34]. Phylogenetic analyses were conducted using the neighbor joining method in MEGA. The following accession numbers were used: AtOxR (GenBank accession number: NP_568854), *Oryza sativa* (NP_001058445), *Glycine max* (NP_001235783), *Zea mays* (ACG39093), *Setaria italica* (XP_004966149), *Medicago truncatula* (XP_003610078), *Eutrema salsugineum* (ESQ42667), *Capsella rubella* (EOA14055), *Theobroma cacao* (EOY31908), and *Ricinus communis* (XP_002532085).

Yeast transformations were performed using a lithium acetate-based method [32]. The empty vector plasmids *pYES2-AtOxR* and *pYES2* were used as controls, and were introduced into the yeast strain InVSCI. The transformed yeast strains were grown in synthetic defined (SD) medium lacking the appropriate amino acids for the selective growth for the expression plasmids. For the response assays, yeast transformants were cultured in liquid yeast extract peptone dextrose (YPD) medium (1 % yeast extract, 2 % peptone, and 2 % glucose) until they reached an optical density at 600 nm of ≈ 0.6, and were then diluted 10^−1^-, 10^−2^-, 10^−3^-,10^−4^-, and 10^−5^-fold using double distilled H_2_O. Then, aliquots of each dilution were spotted onto solid yeast YPD and YPG medium (1 % yeast extract, 2 % peptone, and 2 % galactose) supplemented with different concentrations of NaCl (0.7 M, 0.9 M, 1 M), Mannitol (1 M, 1.2 M, 1.5 M), H_2_O_2_ (4 mM, 4.5 mM, 4.8 mM), MnCl_2_ (1 mM, 1.5 mM), MgCl_2_ (0.8 M, 1 M), CdCl_2_ (160 μM, 180 μM), BaCl_2_ (2 mM, 4 mM), CuCl_2_ (7 mM, 8 mM), FeCl_3_ (7 mM, 10 mM), AlCl_3_ (5.5 mM, 6 mM, 6.5 mM, 7 mM). A yeast transformant of the *pYES2* empty vector was used as a control, and growth was monitored for 3–7 days at 30 °C.

### Analysis of gene expression using quantitative real-time PCR

*Arabidopsis* seeds were surface sterilized and plated on solid half MS medium. After 2 days stratification at 4 °C, the plates were stored in a 22 °C incubator for propagation. The seedlings were transferred from the plates to a 1:1 mixture of soil and vermiculite and grown to maturity at 22 °C. The plants were cultured under a 16-h-light/8-h-dark cycle in a growth chamber. Roots, stems, leaves, panicle, and siliques of 2-month-old plants were sampled for qRT-PCR. A second batch of seedlings were pre-cultured for 2 weeks on 1/2 solid medium, and then treated with different concentrations of various stresses (150 mM NaCl, 300 mM mannitol, 50 μM CuCl_2_, or 3 mM H_2_O_2_), the shoots and roots were sampled after 0 h, 6 h, 12 h, and 24 h treatment and used for qRT-PCR analyses.

Total RNA was isolated using the RNeasy plant Mini kit (Qiagen, Hilden, Germany), and treated with RNase-free DNaseI (Qiagen, Hilden, Germany). First-strand cDNA was synthesized using SuperScript III reverse transcriptase (Invirogen, California, USA). Gene-specific primers pairs AtOxR-RT-FW and AtOxR-RT-RV were used for AtOxR, while Actin-FW and Actin-RV were used for Actin (Table 1). Relative quantification using qRT-PCR reactions were performed with SYBR green I using the LightCycler®480 system II (Agilent, USA).

### Localization analysis of AtOxR protein in yeast and plant cells

Yeast transformants carrying the plasmids *pYES2-AtOxR-GFP* and *pYES2-GFP* were pre-cultured in liquid YPD medium overnight at 30 °C, washed three times with distilled water to remove the glucose and cultured in liquid SD uracil medium with galactose at 30 °C to induce the expression of GFP and AtOxR-GFP under the control of GAL promoter. Live cells were incubated with ER Tracker, and the localization of AtOxR-GFP was observed with a confocal microscope (Olympus Fluoview, FV500). Five-day-old transgenic roots carrying pBI121-GFP and pBI121-AtOxR-GFP were incubated with 1 μM Endoplasmic Reticulum (ER) Tracker for 15 min at 37 °C, and the localization of AtOxR-GFP was observed with a confocal microscope (Olympus Fluoview, FV500, Japan). GFP fluorescence was detected between 505 and 550 nm with excitation at 488 nm, ER Tracker dyes signals were detected using a 615 nm emission filter with excitation at 587 nm.

### Analysis of the transgenic *Arabidopsis* plants

T3 homozygous transgenic plants overexpressing-AtOxR in a Col-0 backgrounds were selected with Kanamycin. Total RNAs of the individual lines were obtained using TRIzol. Denaturing gel electrophoresis was performed to examine the transformation of the *AtOxR* gene, which was identified using a probe labeled with digoxigenin (DIG, Roche, USA) followed by RNA gel blotting according to the methods described by [35]. Signals were detected using a luminescent image analyzer (Fujifilm, LAS-4000mini, Japan). The single lines were named #1, #2, #3, respectively. The seeds of transgenic plants were treated 10 days with different concentrations of NaCl (100 and 125 mM),mannitol (100 and 200 mM),CuCl_2_ (10 and 50 μM) and H_2_O_2_ (1 and 2 mM). T3 Seeds from the *Arabidopsis* Col-0 lines were surface-sterilized, grown on 1/2 MS plates, and supplemented with different concentrations of NaCl (100 and 125 mM) and H_2_O_2_ (1 and 2 mM) for 2 weeks. The seedling were grown under 16 h/8 h light/dark cycles at 22 °C, the root lengths were measured. Statistical analyses were performed using Student’s *t*-tests.

### H_2_O_2_ content measurement

The H_2_O_2_ content was measured in 10-day-old WT and T3 generation transgenic plants that overexpress AtOxR and that had been treated with 150 mM NaCl, 300 mM mannitol, 50 μM CuCl_2_, and 3 mM H_2_O_2_ for 12 h, 24 h, or 48 h. Approximately 0.1 g fresh weight of each sample was harvested and immediately ground in liquid nitrogen with a mortar and pestle. The H_2_O_2_ content was measured colorimetrically at 415 nm by the titanium tetrachloride reaction method [36].

### Measurement of total AsA content

To measure the AsA levels, seedlings of WT and T3 generation transgenic *A.thaliana* were grown on MS medium for 10 d, and that had been treated with 150 mM NaCl, 300 mM mannitol, 50 μM CuCl_2_, and 3 mM H_2_O_2_ for 12 h, 24 h, or 48 h. Approximately 0.1 g fresh weight of each sample was harvested and immediately ground in liquid nitrogen with a mortar and pestle. The AsA content was determined using an AsA content test kit (Comin, Soochow, China). Briefly, samples were ground under liquid nitrogen and homogenized in 1 mL of cold extraction buffer (solution I). The homogenate was centrifuged at 10000 rpm for 20 min at 4 °C, then the supernatant or standard solution (100 μl) were incubated with 800 μl solution II and 100 μl solution III, respectively, and pipetting immediately at room temperature. Then, the absorption values of 30 s and 150 s at 265 nm with UV spectrophotometer were used for calculated the total AsA levels of plants. Three biological replications were used for statistical analyses. Statistical significance was determined using Student’s *t*-tests.

## Results

### *AtOxR*, a single copy gene encoding an ER protein in *Arabidopsis*

**Fig. 1:**
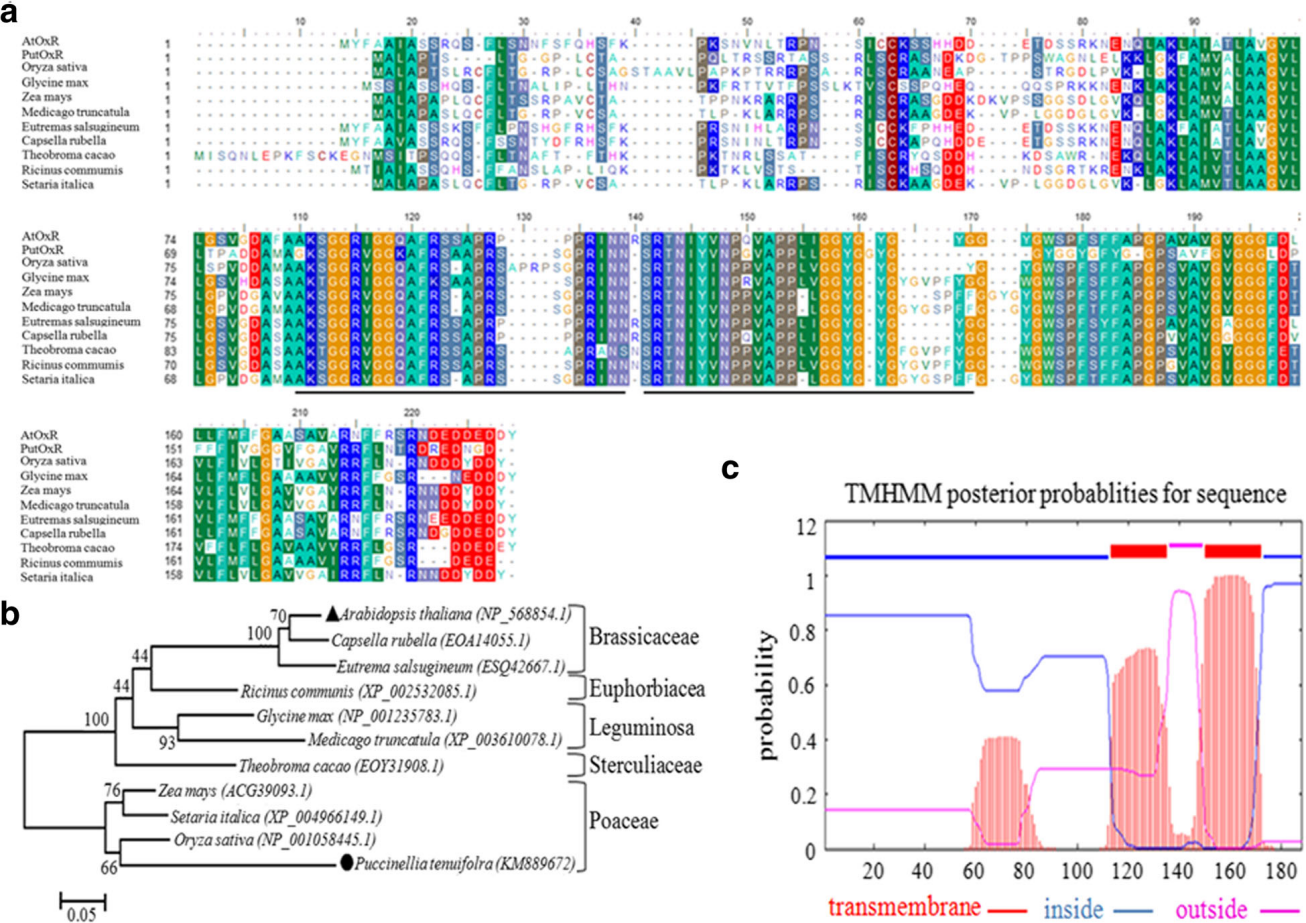
Sequence and bioinformatic analyses of *AtOxR*. (**a**) Alignment of the amino acid sequence of AtOxR from *Arabidopsis thaliana* (GenBank No. NP_568854) with those of *Oryza sativa* (NP_001058445), *Glycine max* (NP_001235783), *Zea mays* (ACG39093), *Setaria italica* (XP_004966149), *Medicago truncatula* (XP_003610078), *Eutrema salsugineum* (ESQ42667), *Capsella rubella* (EOA14055), *Theobroma cacao* (EOY31908), and *Ricinus communis* (XP_002532085). **b** Phylogenetic trees based on the amino acid sequence of AtOxR and homologous sequences from the GenBank database. The accession numbers are listed in the Experimental Procedures section. (**c**) Transmembrane domains in AtOxR were predicted by the TMHMM algorithm (http://www.cbs.dtu.dk/services/TMHMM/), and are underlined in black in (**a**)

A BLAST search of the NCBI database for matches to *PutOxR* identified one candidate (GenBank accession NO: NP_568854), which we named AtOxR. The sequence is from a gene of unknown function with 61 % identity to *PutOxR* at the amino acid sequence level. *AtOxR* is a single copy gene with a 564-bp open reading frame (ORF) encoding 188 amino acids with a predicted molecular mass of 20.19 kDa. Homologous proteins are found in several other plants (Fig. 1a). In a phylogenetic tree based on the amino acid sequences of the conserved region, AtOxR is most closely related to *Eutrema salsugineum* and *Capsella rubella* of the family Brassicaceae (Fig. 1b). AtOxR is predicted to have two transmembrane domains by the TMHMM algorithm (Fig. 1a, c).

**Fig. 2:**
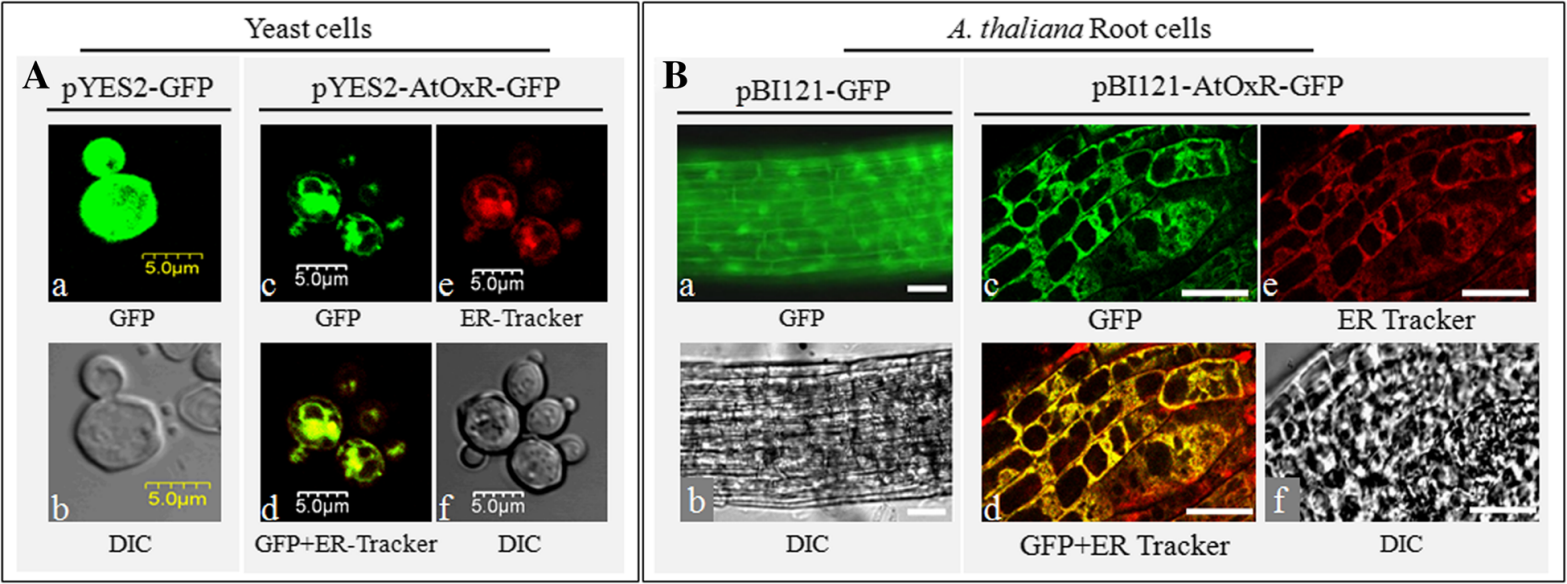
Subcellular localization of AtOxR-GFP. **A** Expression of GFP protein (a, b) and AtOxR-GFP fusion constructs (c~f) in yeast cells. Scale bars = 5 μm. **B** Expression of GFP protein (a, b) and AtOxR-GFP fusion constructs (c~f) in *A. thaliana * root cells. The GFP column shows the signal detected in the green channel; the ER Tracker column shows the signal detected in the *red* channel; the GFP+ ER Tracker column corresponds to the merging of the *green* and *red* channels, in which *yellow* represents the superposition of *green* and *red*. DIC: differential interference contrast. Scale bars = 20 μm

The ER was visualized using ER Tracker, an ER-specific dye for living cells. Because eukaryotic yeast shares similar cellular structures with plant cells, the subcellular localization of AtOxR in yeast and plant cells were both analysed with an ER Tracker in this study. We observed that the green fluorescence of GFP alone was almost evenly distributed throughout the yeast (Fig. 2A-a) and *Arabidopsis* root cells (Fig. 2B-a), whereas the green fluorescence of AtOxR-GFP fusion protein (Fig. 2A-c, B-c) and the red fluorescence of the ER Tracker (Fig. 2 A-e, B-e) overlapped (Fig. 2 A-d, B-d) in both yeast cells and *Arabidopsis* root cells (Fig. 2). These results suggested that the AtOxR-GFP was localized in the ER in yeast and *Arabidopsis* cells (Fig. 2), *AtOxR* is a single copy gene ecoding an ER protein in *Arabidopsis* genome.

### Gene expression of AtOxR is induced by abiotic stresses in *Arabidopsis*

**Fig. 3:**
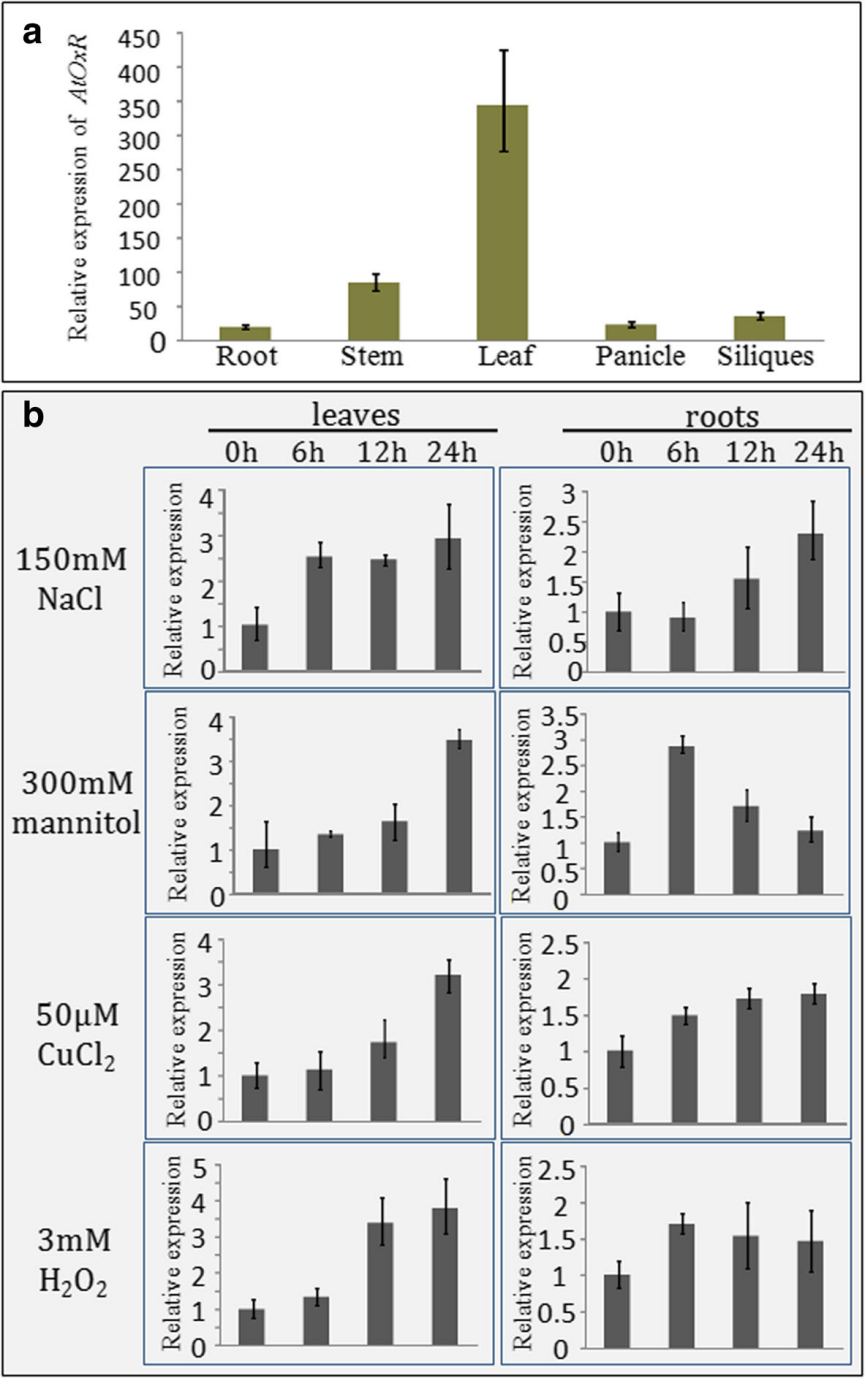
Expression analysis of *AtOxR* gene from *A. thaliana*. (**a**) Quantitative RT-PCR analysis of *AtOxR* in different organs of *A. thaliana*. (**b**) Quantitative RT-PCR analysis of the expression of *AtOxR* in response to various abiotic stresses (see Methods for details). *AtOxR* expression was normalized against *Actin* mRNA levels; the reported data are the means of three replicate experiments ± S.E

To examine the biological functions of *AtOxR*, we first investigated its expression pattern in various organs by qRT-PCR. This analysis showed that *AtOxR* was expressed in all *Arabidopsis* plant organs (root, stem, leaf, panicle, and siliques), with the highest levels of expression in leaves under normal conditions (Fig. 3a). When plants were grown in the presence of 150 mM NaCl, *AtOxR* mRNA expression, as shown by qRT-PCR, was induced within 6 h of treatment, declined at 12 h, and then increased again after 24 h in the leaves. In roots, *AtOxR* mRNA was slightly declined at 6 h after treatment, and then increased gradually and peaked at 24 h. In the presence of 300 mM mannitol, *AtOxR* mRNA expression peaked at 24 h in leaves, whereas expression was induced at 6 h after inititation of treatment in roots, and then began to decline gradually. After stressing with 50 μM CuCl_2_, *AtOxR* mRNA expression peaked at 24 h in both the leaves and roots. In the presence of 3 mM H_2_O_2_, *AtOxR* mRNA expression peaked at 24 h in both the leaves; in contrast, expression peaked at 6, and then declined in roots (Fig. 3b). Overall, these results suggest that the *AtOxR* gene confers a response to multiple abiotic stresses in both leves and roots of *Arabidopsis*.

### Overexpression of *AtOxR* gene enhances tolerance to abiotic stresses in yeast and *Arabidopsis*

**Fig. 4:**
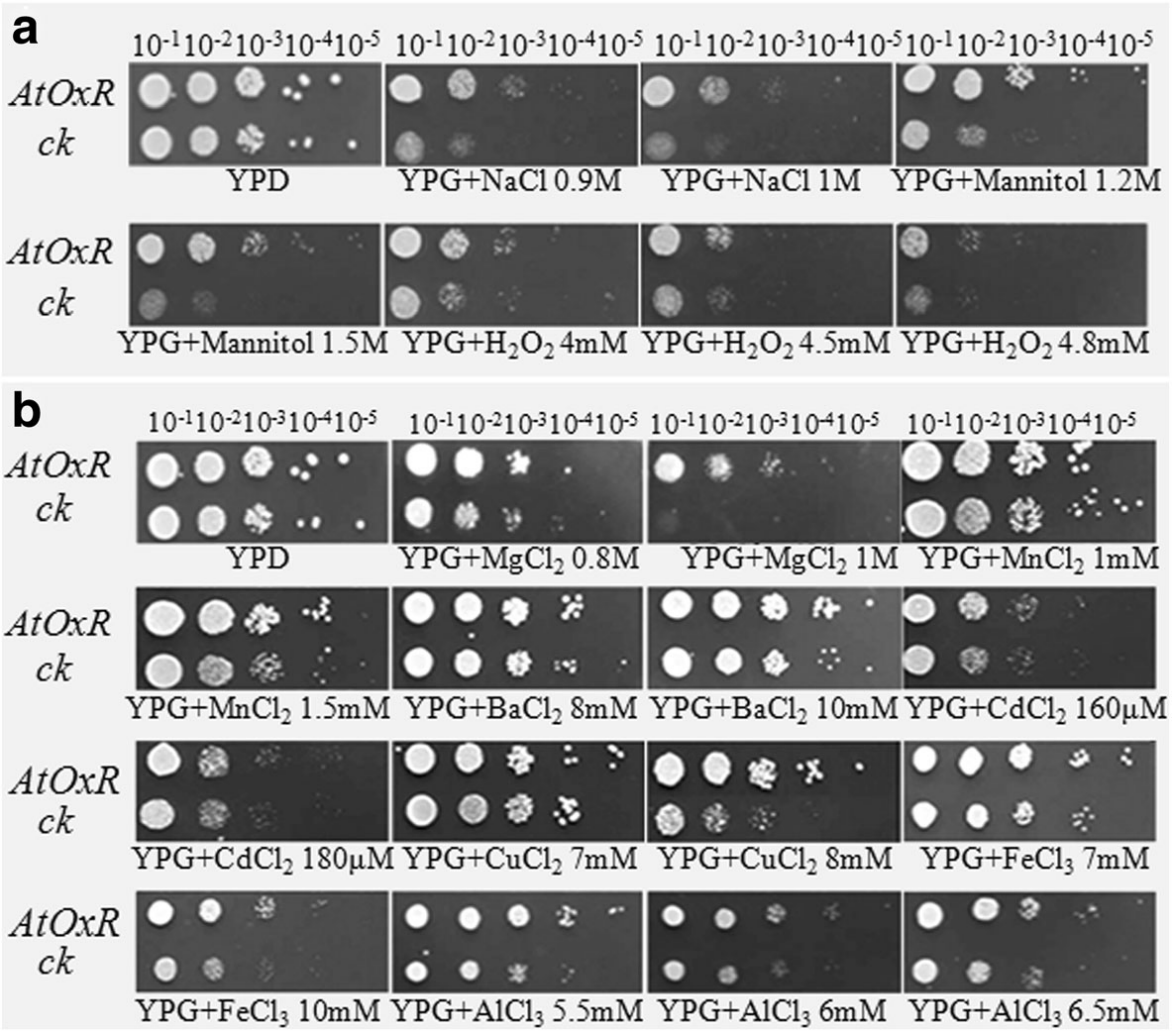
Growth of AtOxR yeast transformants in response to a variety of abiotic stresses. Yeast transformants were grown on (**a**) YPD medium containing NaCl (0 mM, 0.7 mM, 0.9 mM, and 1 mM), mannitol (0 mM, 1 mM, 1.2 mM, and 1.5 mM), and H_2_O_2_ (0 mM, 4 mM, 4.5 mM, and 4.8 mM) in the presence of 2 % (w/v) galactose and (**b**) YPD medium containing different concentrations of the following metal ions: MgCl_2_ (0.8 mM and 1 mM), MnCl_2_ (1 mM and 1.5 mM), BaCl_2_ (8 mM and 10 mM), CdCl_2_ (160 μM and 180 μM), CuCl_2_ (7 mM and 8 mM), FeCl_3_ (7 mM and 10 mM), AlCl_3_ (5.5 mM, 6 mM, 6.5 mM, and 7 mM) in the presence of 2 % (w/v) galactose. Growth was monitored for 3–6 days at 30 °C

Growth of transgenic yeast cells carrying AtOxR was better on solid yeast YPG medium than that of control cells on media containing NaCl, mannitol, or H_2_O_2_ (Fig. 4a). The transformants also grew much better than did the empty vector transformants on media containing Al^3+^, Mg^2+^, Cu^2+^,Mn^2+^, Ba^2+^, or Fe^3+^ (Fig. 4b), suggesting that AtOxR is associated with the oxidative stress caused by high levels of cations.

**Fig. 5:**
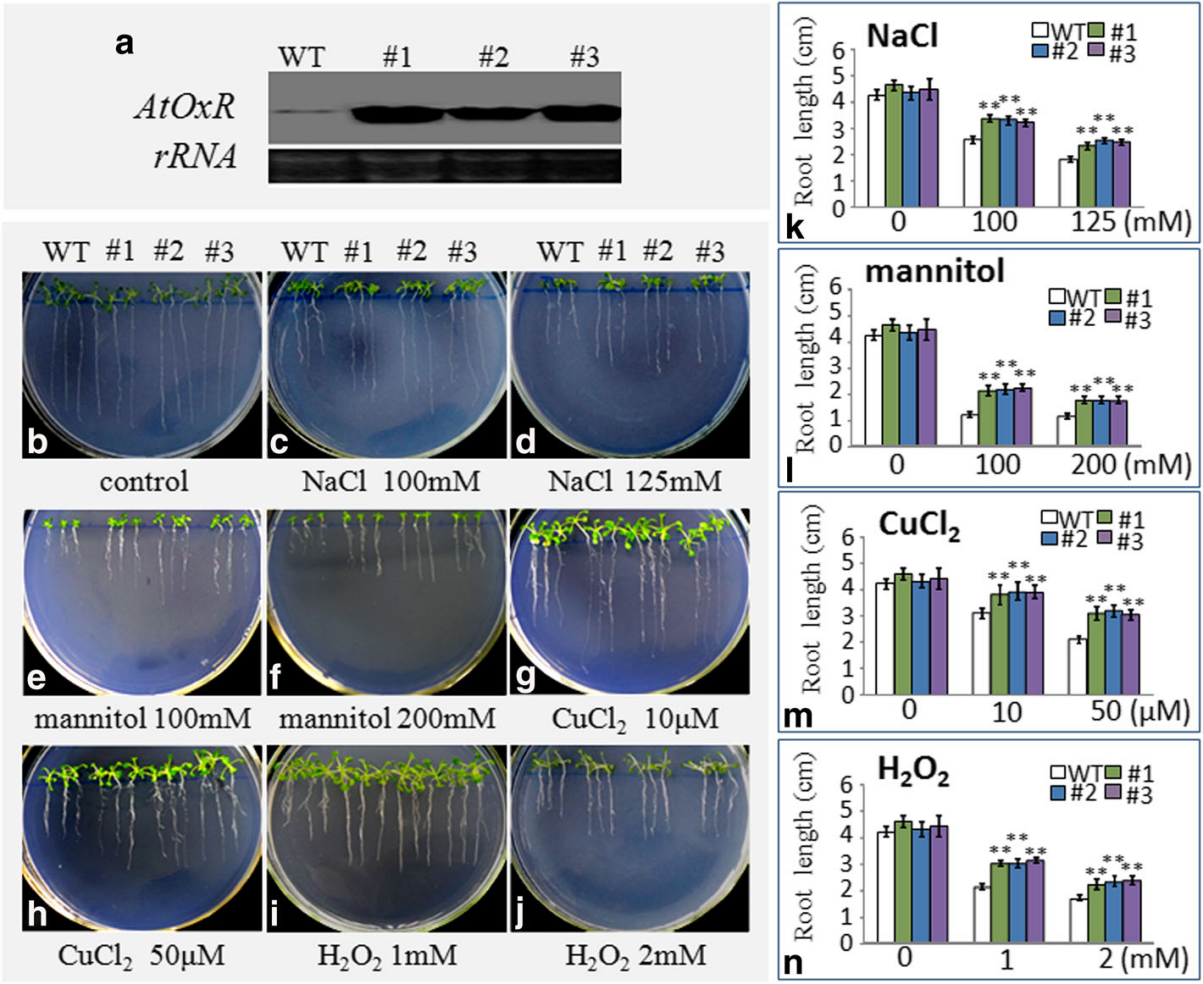
Relative abiotic stress tolerance of wild-type (WT) and *AtOxR*-transgenic plants. (**a**) RNA gel blot analysis of T3 transgenic plants expressing AtOxR. WT: *Arabidopsis thaliana* ecotype Columbia-0; #1, #2, and #3: T3 seedlings with AtOxR on a Columbia-0 background. (**b**–**j**) Growth of WT plants and transgenic AtOxR plants (#1–3) on medium containing 1/2 MS (B), 100 and 125 mM NaCl (**c**–**d**), 100 and 200 mM Mannitol (**e**–**f**), 10 and 50 μM CuCl_2_ (**g**–**h**), and 1 and 2 mM H_2_O_2_ (**i**–**j**) for 14 days. (**k**–**n**) Root lengths of WT and AtOxR transgenic plants were measured after treatment. Each value represents the means ± SE of 15 plants. Statistical significance was determined using Student’s *t*-tests. *represents *p* < 0.05 and **represents *p* < 0.01

Three *Arabidopsis* transgenic plants that overexpressed AtOxR under the control of the CaMV35S promoter (#1–3) were identified by northern blotting (Fig. 5a). Control samples showed weak AtOxR signals, whereas the transgenic plants had higher expression confirming that the plants had been successfully transformed with AtOxR. In seedlings exposed to NaCl (100 and 125 mM), mannitol (100 and 200 mM), CuCl_2_ (10 and 50 μM), or H_2_O_2_ (1 and 2 mM), both the primary roots and leaves grew better in the AtOxR transgenic lines compared to WT plants (Fig. 5c–j), whereas the growth of WT was similar to that of the transgenic lines on control medium (Fig. 5b). Measurements confirmed that the root lengths (Fig. 5k–n) of AtOxR transgenic lines were higher than those of WT plants under stress conditions. These results suggest that overexpression of AtOxR improved the tolerance to multiple abiotic stresses in *Arabidopsis*.

### Determination of H_2_O_2_ and AsA content in AtOxR transgenic *Arabidopsis* plants under abiotic stresses

**Fig. 6:**
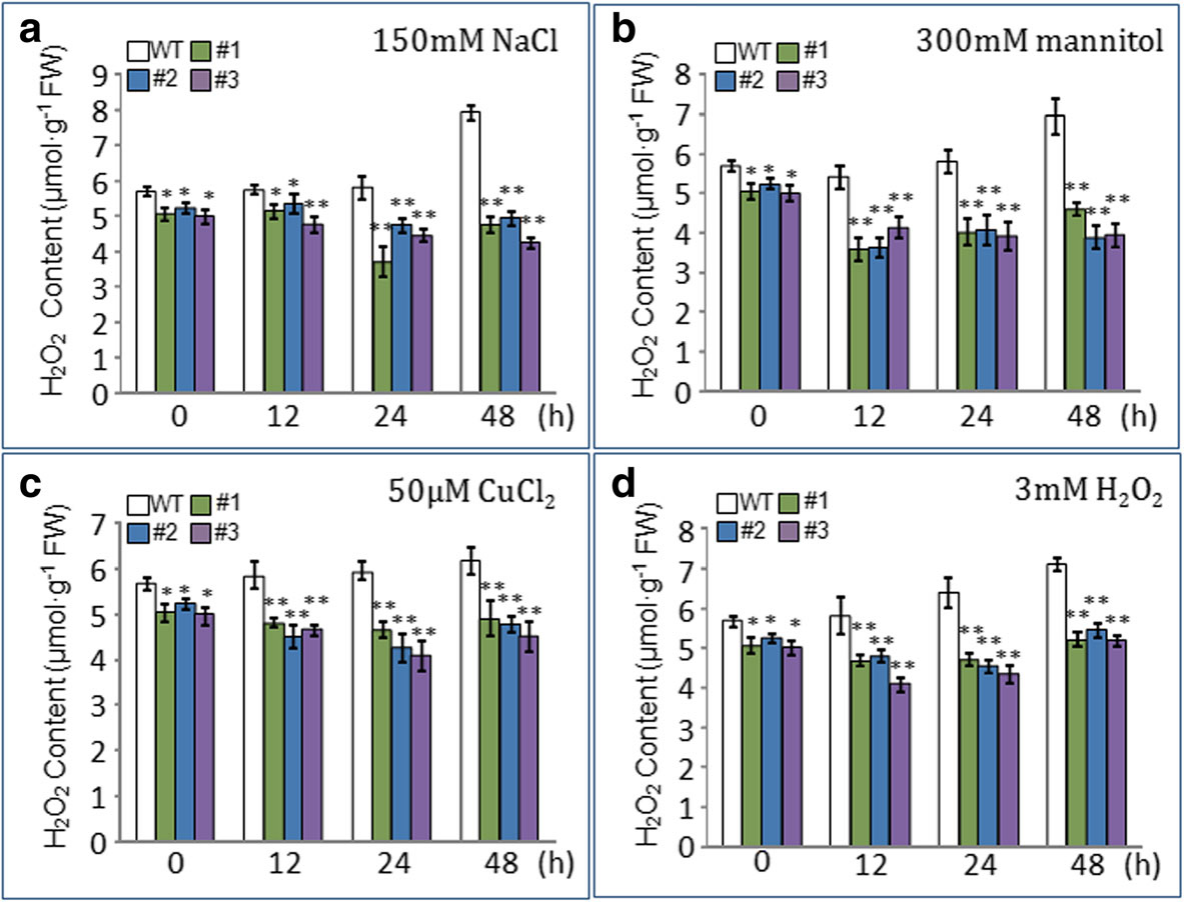
Effect of abiotic stresses on H_2_O_2_ content in WT and transgenic plants. The H_2_O_2_ content of 10-day-old WT and T3 generation transgenic plants overexpressing AtOxR were assessed after 12, 24, or 48 h of treatment: (**a**) 150 mM NaCl; (**b**) 300 mM mannitol; (**c**) 50 μM CuCl_2_; (**d**) 3 mM H_2_O_2_. The means ± standard deviations (SDs) of three replicates are shown. Statistical significance was determined using Student’s *t*-tests. *represents *p* < 0.05 and **represents *p* < 0.01

On the control medium, the H_2_O_2_ contents were slightly lower in three transgenic plants than those of WT plants, whereas H_2_O_2_ levels began to significant decrease in the three transgenic plants within 12 h of exposure to 150 mM NaCl, 300 mM mannitol, 50 μM CuCl_2_, or 3 mM H_2_O_2_, comparable to that of WT plants (Fig. 6). These results suggest that AtOxR is associated with H_2_O_2_ scavenging to lower oxidative stress.

**Fig. 7:**
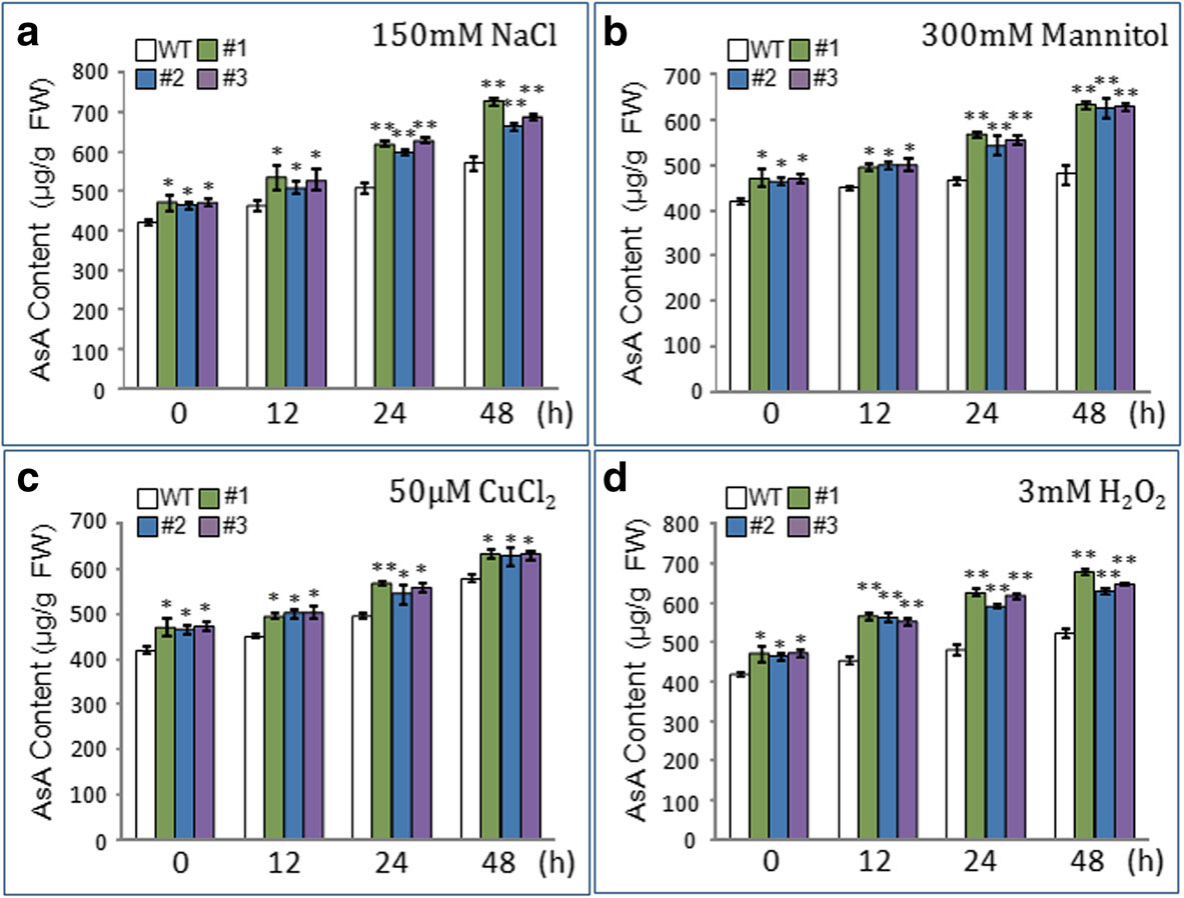
Effect of abiotic stresses on AsA content in WT and transgenic plants. AsA content of 10-day-old WT and T3 generation transgenic plants overexpressing AtOxR were assessed after 12 h, 24 h, or 48 h treatment: (**a**) 150 mM NaCl; (**b**) 300 mM mannitol; (**c**) 50 μM CuCl_2_; (**d**) 3 mM H_2_O_2_. The means ± SDs of three replicates are shown. Statistical significance was determined using Student’s *t*-tests. * represents *p* < 0.05 and ** represents *p* < 0.01

In addition, we also test the AsA content in AtOxR transgenic plants under abiotic stresses conditions. Anylsis the phenotype of AsA levels in the normal condition (absence of stresses), the AsA content was higher in the transgenic plants than in the WT, while in the presence of stresses (150 mM NaCl, 300 mM mannitol, 50 μM CuCl_2_, and 3 mM H_2_O_2_), it was significantly higher after 12 h, 24 h, and 48 h treatment (Fig. 7a–d). To further confirm the content of AsA in WT and AtOxR transgenic plants under abiotic stresses conditions, HPLC method was used to determine the total AsA content in the tissues under 150 mM NaCl and 3 mM H_2_O_2_ conditions for 24 and 48 h treatment. The HPLC analysis showed similar trend to the results of test kit analysis (see Additional file 1). Overall, these results suggest that overexpression of AtOxR improves the accumulation of AsA content in transgenic plants compared with WT plants under abiotic stress conditions.

## Discussion

AsA is known to play a role in response to oxidative stress, although the regulatory molecular mechanism of AsA synthesis has not been yet well understood. Here, we showed that *AtOxR*, an *Arabidopsis* gene of unknown function, is related to the levels of AsA and H_2_O_2_. AtOxR is predicted to have two transmembrane domains. AtOxR-GFP was localized to the ER in yeast and *Arabidopsis* cells (Fig. 2), the localization pattern of AtOxR-GFP is agreement with previous reports [37, 38]. Treatment with NaCl, mannitol, metal ions, or H_2_O_2_ induced *AtOxR* expression in both the leaves and roots (Fig. 3b). In plants, salt, osmotic stress, and metal ions can be indirect produce reactive oxygen species (ROS), which leads to oxidative stress [39–41]. Together, these findings suggest that the expression of AtOxR might be associated with oxidative stress. In addition, overexpression of *AtOxR* in yeast and *Arabidopsis* enhanced their tolerances to multiple abiotic stresses (Figs. 4 and 5). High levels of cations (such as Al^3+^, Cu^2+^, and Fe^3+^), salt, osmotic stress, etc. can be indirect produce ROS, which lead to oxidative stress [39, 41, 42]. Overexpressing AtOxR also improved the tolerance to metal ions in yeast and *Arabidopsis* (Figs. 4 and 5). Thus, these results suggest that the *AtOxR* gene has a role in the response to multiple abiotic stress, and associated with oxidative stress in yeast and plants.

Although moderate levels of ROS have a role in regulating various biological processes such as hormone signaling, and biotic and abiotic stress responses [1, 43], excessive ROS can cause irreversible damage in plants [3–5]. Our finding that AtOxR-overexpressing transgenic plants accumulated less H_2_O_2_ than did WT after challenge with NaCl, mannitol, CuCl_2_, and H_2_O_2_ (Fig. 6) is similarly to previous reports that the overexpression of stress resistance-related genes resulted in less H_2_O_2_ accumulation in response to abiotic stresses [14, 44–47].

Plants have four major H_2_O_2_-scavenging pathways. Two of these pathways, the water-water cycle and the ascorbate-glutathione cycle [3], are related to AsA, which is known to have roles in plant stress responses [48, 49]. Therefore, the effect of AtOxR on conferring tolerance to multiple stresses might be caused by the increased AsA content in AtOxR transgenic plants. In addition, AsA also has a role in ROS detoxification, as an antioxidant and H_2_O_2_-scavenger in plant cell to avoid accumulation of ROS under stress conditions [3, 50, 51]. In our study, the increased AsA content in AtOxR-expressing *Arabidopsis* in response to abiotic stresses (Fig. 7 and Additional file 1) suggests that AtOxR improves tolerance to abiotic stresses by increasing the AsA, which in turn promotes the scavenging of excess H_2_O_2_. Similar result was obtained in the transgenic plants expressing *DHAR*, *GalDH* and co-expression of *NCED* and *ALO* increased tolerance to abiotic stresses with elevated levels of AsA [28, 31, 52]. Therefore, our result suggest that overexpression of AtOxR is an effective way for use in crops improvement for increased tolerance to abiotic stresses and nutrition quality.

## Conclusions

In this study, our results suggest that expression of *AtOxR* gene is response to abiotic stresses in roots and leaves of *Arabidopsis*. The H_2_O_2_ and AsA content of AtOxR-overexpressing transgenic plants were significantly lower and significantly higher, respectively, than those of WT plants under abiotic stress conditions, furthermore, overexpression of *AtOxR* gene improves abiotic stresses tolerance in *Arabidopsis*. In addition to this, because AsA is an important component of human nutrition, modified expression of AtOxR offers potential for the development of crop varieties with elevated AsA. Hence, increasing the AsA content in crops is important for further agriculture production through moleculer breeding.

## Additional file

Additional file 1: Total AsA content in WT and transgenic plants were performed by HPLC analysis. AsA content of 10-day-old WT and T3 generation transgenic plants overexpressing AtOxR were assessed after 24 h, or 48 h treatment: (A) 150 mM NaCl; (B) 3 mM H_2_O_2_. The clear extracts (10 μL) were injected directly into the HPLC instrument (RIGOL L-3000), and chromatographic separation was achieved on an Sepax GP-C18 (250 × 4.6 mm, 5 mm) column and detected at 254 nm with a UV detector. The means ± SDs of three replicates are shown. Statistical significance was determined using Student’s *t*-tests. * represents *p* < 0.05 and ** represents *p* < 0.01. (TIF 1460 kb)

## Abbreviations

APX: Ascorbate peroxidase
AsA: L-ascorbic acid
CAT: Catalase
DIG: Digoxigenin
ER: Endoplasmic reticulum.
GPX: Glutathione peroxidase
GSH: Glutathione
H_2_O_2_: Hydrogen peroxide
MS: Murashige and skoogmedium
O_2_^−^: Superoxide anions
OH^−^: Hydroxyl radicals
qRT-PCR: Quantitative real-time PCR
ROS: Reactive oxygen species
SD: Synthetic defined medium
SOD: Superoxide dismutase
YPD: Yeast extract peptone dextrose medium
YPG: Yeast extract peptone galactose medium
