# Supplementary figures and images for: Overexpression of AtOxR gene improves abiotic stresses tolerance and vitamin C content in Arabidopsis thaliana
Source: BMC Biotechnol. 2016 Oct 7;16:69. doi: 10.1186/s12896-016-0299-0 (PMC5055693; doi:10.1186/s12896-016-0299-0)

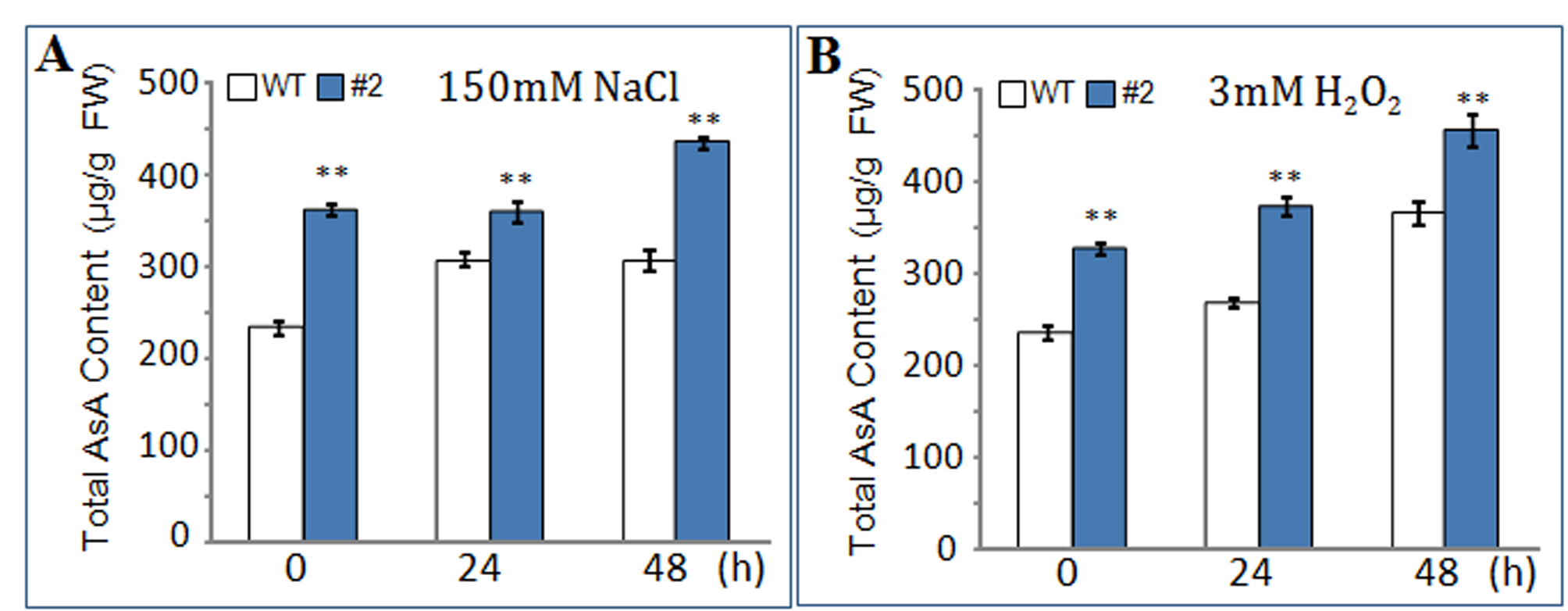

Supplement: Additional file 1: — Total AsA content in WT and transgenic plants were performed by HPLC analysis. AsA content of 10-day-old WT and T3 generation transgenic plants overexpressing AtOxR were assessed after 24 h, or 48 h treatment: (A) 150 mM NaCl; (B) 3 mM H2O2. The clear extracts (10 μL) were injected directly into the HPLC instrument (RIGOL L-3000), and chromatographic separation was achieved on an Sepax GP-C18 (250 × 4.6 mm, 5 mm) column and detected at 254 nm with a UV detector. The means ± SDs of three replicates are shown. Statistical significance was determined using Student’s t-tests. * represents p < 0.05 and ** represents p < 0.01. (TIF 1460 kb) [file 12896_2016_299_MOESM1_ESM.tif]
